# Analysis of User-Generated Posts on Social Media of Adjuvant Analgesics: A Machine Learning Study

**DOI:** 10.7150/ijms.96981

**Published:** 2025-01-01

**Authors:** Federico Carabot, Carolina Donat-Vargas, Francisco J. Lara-Abelenda, Oscar Fraile- Martínez, Javier Santoma, Cielo Garcia-Montero, Teresa Valadés, Luis Gutierrez-Rojas, MA Martinez-González, Miguel Angel Ortega, Melchor Alvarez-Mon, Miguel Angel Alvarez-Mon

**Affiliations:** 1 Department of Medicine and Medical Specialities. University of Alcala, Alcala de Henares, 28801 Madrid, Spain.; 2 Ramón y Cajal Institute of Sanitary Research (IRYCIS), 28034 Madrid, Spain.; 3 ISGlobal, Barcelona, Spain.; 4 CIBER Epidemiología y Salud Pública (CIBERESP), Madrid, Spain.; 5 Cardiovascular and Nutritional Epidemiology, Unit of Institute of Environmental Medicine, Karolinska In-stitute, Stockholm, Sweden.; 6 Departamento Teoria de la Señal y Comunicaciones y Sistemas Telemáticos y Computación, Escuela Tecnica Superior de Ingenieria de Telecomunicación, Universidad Rey Juan Carlos, 28942 Fuenlabrada, Spain.; 7 Department of Psychiatry and CTS-549 Research Group, Institute of Neurosciences, University of Granada, Granada, Spain.; 8 Department of Psychiatry, San Cecilio University Hospital, Granada, Spain.; 9 Centro de Investigación Biomédica en Red Fisiopatología de la Obesidad y la Nutrición (CIBEROBN), Insti-tute of Health Carlos III, Madrid, Spain.; 10 Department of Preventive Medicine and Public Health, Instituto de Investigación Sanitaria de Navarra (IdiSNA), University of Navarra, Pamplona, Spain.; 11 Department of Nutrition, Harvard T.H. Chan School of Public Health, Boston, MA, USA.; 12 Department of Psychiatry and Mental Health. Hospital Universitario Infanta Leonor, Madrid, Spain.; 13 CIBERSAM-ISCIII (Biomedical Research Networking Centre in Mental Health), Madrid, Spain.

**Keywords:** Machine Learning, Pain, Coanalgesics, X social media, Twitter

## Abstract

**Background:** Antiepileptics and antidepressants are frequently prescribed for chronic pain, but their efficacy and potential adverse effects raise concerns, including dependency issues. Increased prescriptions, sometimes fraudulent, prompted reclassification of antiepileptics in some countries. Our aim is to comprehend opinions, perceptions, beliefs, and attitudes towards co-analgesics from online discussions on X (formerly known as Twitter), offering insights closer to reality than conventional surveys.

**Methods:** In this cross-sectional study, we collected 77,183 public posts about co-analgesics in English or Spanish from January 1^st^ 2019 to December 31st, 2020. A total of 51,167 post were included, and 2,000 were manually analyzed using a researcher-created codebook. Machine learning classifiers were then applied to the remaining datasets to determine the number of publications for each user type and identify categories through content analysis.

**Results:** Of the 51,167 posts analyzed, 78% discussed anticonvulsants and 24% discussed analgesic antidepressants (Percentages add up to more than 100% because there were 1,300 posts containing references to both types of medications). Only 13% were authored by healthcare professionals, while 67% were from patients. Medical content predominated, with 70% noting low medication efficacy and almost 50% referencing side effects. Non-medical content included challenges in dispensing (25%), complaints about high costs (15%), and trivialization of medication use (10%).

**Conclusions:** This study offers valuable insights into public perceptions of co-analgesics. Findings aid in designing public health communications to raise awareness of associated risks, urging both healthcare providers and the public to optimize drug use.

## Introduction

Chronic pain is a common reason for seeking medical attention and often requires a multifactorial approach to management. Opioids are the cornerstone of pain treatment but have serious long term side effects 1,2. Their misuse has created a huge public health problem in many countries, particularly in the United States, with a heavy death toll 3. The inappropriate use of this medication is also associated with increased rates of anxiety and depressive disorders 3.

Therefore, it is common to use adjunctive medications such as pregabalin, gabapentin, and tricyclic antidepressants. In fact, there has been an increasing use and prescription of these adjunctive medications 4,5. Studies have shown a rise in amitriptyline prescriptions for purposes other than depression 6, with antidepressants being the most commonly used for off-label indications 7. Amitriptyline was the third most prescribed antidepressant in Germany in 2008 and the second most prescribed antidepressant in England between 2015 and 2019 8-10. In Canada, almost half (48.4%) of amitriptyline prescriptions are for pain relief 11. The use of gabapentinoids in the United States has more than tripled between 2002 and 2015, with a 50% increase in prescriptions between 2012 and 2016 12, and the number of prescriptions continues to rise in the USA 13. In the United Kingdom, the use of pregabalin increased by 350% between 2008 and 2013, and the rate of patients newly treated with gabapentinoids tripled from 2007 to 2017 in primary care 14, and nowadays the figures seem to be stabilizing 15,16.

However, caution must be exercised when using these adjunctive medications, as they have multiple pharmacological interactions and are often used in combination with other drugs 17,18. They are also associated with relatively common side effects such as dizziness, drowsiness, difficulties in memory or concentration, nausea, and vomiting 10,19. Furthermore, there is an increasing off-label use of these medications, which is concerning 5,20-22. Tricyclic antidepressants in particular are commonly used in suicide attempts 23.

Moreover, the recreational use of tricyclic antidepressants and anticonvulsants has been well documented, and there has been an alert about the increasing prescriptions 24-26 and misuse, especially of pregabalin 27,28. While pregabalin is considered a controlled substance by the FDA, indicating potential for abuse, it is widely perceived as having minimal abuse liability by patients 29. In France, pregabalin has become the most frequently included drug in counterfeit prescription forms submitted to pharmacies, as reported in the 2019 OSIAP survey 30.

On the other hand, there are many patients who are correctly prescribed these adjunctive medications, but due to their negative beliefs about the treatment patients tend to not adhere properly 31,32. In this context, intentional non-adherence has been described in patients with chronic disease, often attributed to feeling well and deciding not to take the medication due to fear of side effects 33,34. Therefore, it is important to understand the opinions, perceptions, beliefs, and attitudes that patients, healthcare professionals, and society most frequently maintain towards these medications used in pain treatment. Analyzing spontaneous online discussions, such as those on social media platforms, can provide insights closer to reality than traditional survey methods 35,36. In fact, this type of analysis is also being used for pharmacovigilance purposes 37-39. Moreover, con-versations on social media are generated in a more informal and spontaneous environment, making it more likely to reflect people's true beliefs 40-42. Finally, analyzing these posts allows us to understand the opinions of the general population, including patients who are reluctant to participate in conventional surveys, as well as their family members or acquaintances 43-45.

In this infodemiology study, we utilized the social network X, formerly and more commonly known as Twitter. We aimed to: 1) Quantify the frequency of communications on Twitter about adjunctive medications for pain relief (i.e., co-analgesics) and the interest they generate; 2) Characterize the types of users of co-analgesics participating in these conversations; 3) Identify the main thematic content of the posts related to co-analgesics, with a focus on detecting online sales or other activities that may be detrimental to health.

## Materials and Methods

### Twitter search and data collection strategy

**Figure 1:**
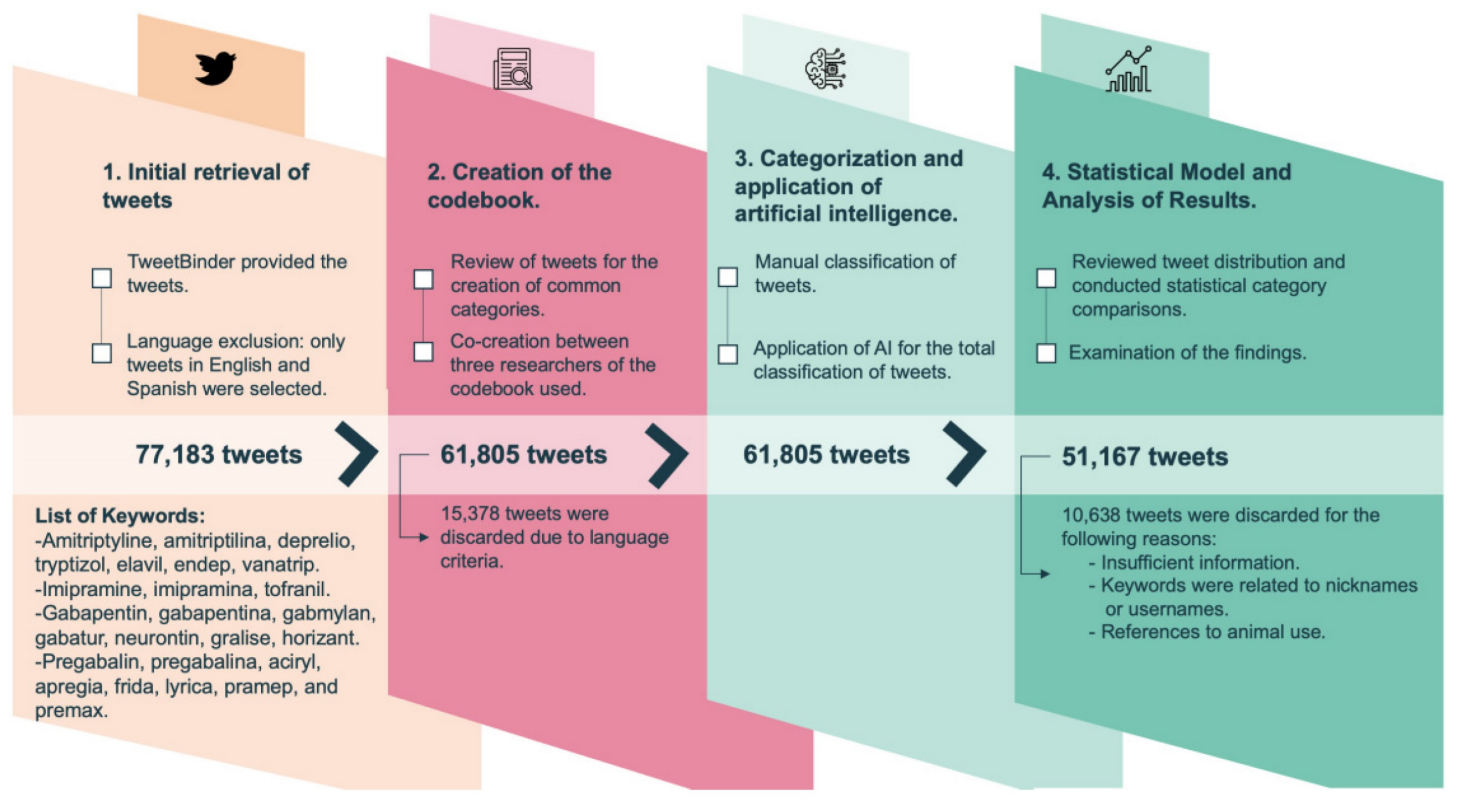
Flowchart.

In this quantitative and qualitative observational study, we focused on searching for posts that referenced co-analgesics. We gathered all publicly available posts utilizing a set of keywords derived from the brand names and generic names of the most commonly prescribed active constituents of pregabalin, gabapentin, amitriptyline, and imipramine in both the United States and Spain. The keywords included were: amitrytiline, amitriptilina, deprelio, tryptizol, elavil, endep, vanatrip, imipramine, Imipramina, tofranil, gabapentin, gabapentina, gabmylan, gabatur, neurontin, gralise, horizant, pregabalin, pregabalina, aciryl, apregia, frida, lyrica, pramep, and premax (Figure 1).

The inclusion criteria for posts were as follows: 1) Published from an open account; 2) Including any of the listed keywords in the post; 3) Posted between January 2019 and December 2020; 4) Written in either Spanish or English. We also collected supplementary data for the posts: the number of retweets and likes generated by each post, as well as the profile description of the users. The tool used for post collection was Tweet Binder, which allows access to 100% of public posts.

### Content analysis process

A total of 77,183 posts were collected, of which 15,378 posts were excluded for being written in a language other than English or Spanish (Figure 1). A codebook was created to analyze the posts. The codebook was completed after the discussion and analysis of 300 posts by 3 researchers. In the codebook, the type of user is the first classified domain, followed by distinguishing between medical and non-medical content. Additionally, we identified posts that posed questions. Regarding the type of user, we distinguished between patients, family members and friends, healthcare professionals, or institutions. To determine the type of user, we examined the profile and the content of the post itself.

Regarding the content, if it was medical in nature, we classified it according to whether it referred to the good or poor efficacy of a drug and whether it mentioned adverse effects. Finally, we classified fake medical content. A paramount consideration was the meticulous specificity of medical content categories. Additionally, in the non-medical content, we distinguished four themes: 1) Management issues; 2) Economic aspects; 3) Solidarity; and 4) Trivialization. Under the category of management issues, we discerned bureaucratic, commercial, and legal content related to pharmacy delivery, medical appointments, commercial aspects, and legal facets. Within the realm of economic considerations, we ascertained whether it pertained to the cost of the pharmaceutical agent.

We grouped the posts by their keywords into 2 categories. The anticonvulsants category contained posts corresponding to pregabalin or gabapentin, and the tricyclics category contained posts corresponding to imipramine and amitriptyline. We selected a total of 1,000 posts for each category, which were manually classified according to the discussed codebook. When the content did not provide sufficient information, when the username matched a keyword, or when the post referred to the use of the drug in animals, it was classified as unclassifiable. Unclassifiable posts were excluded from the study.

### Machine-learning classifier

Technological advances in recent years have allowed the development of multiple emerging scientific disciplines, among them artificial intelligence (AI). AI refers to the development of computer systems that can perform tasks typically requiring human intelligence. It involves the creation of algorithms and models that enable machines to learn from data, reason, recognize patterns, and make decisions autonomously 46. Within AI we can find several branches and one of them is Machine Learning, ML whose objective is to create computational models that extract knowledge from data with a reasonable capacity for generalization. Finally, within ML you can find Deep Learning (DL). DL uses models called neural networks, which are AI methods inspired by human brain neurons whose function is to process information 47. Neural networks have multiple applications ranging from market prediction 48, through detection of infections 49 or the detection of faces in images 50.

Inside ML we are able to find, another type of differentiation depending on the type data used to train the model: supervised, unsupervised and semi-supervised Learning 51. In supervised learning, algorithms are trained with labeled data and they learn patterns to make predictions on new data. Unsupervised learning involves training the algorithm on unlabeled data to identifies inherent patterns or clusters. Lastly, semi-supervised learning is a combination of supervised and unsupervised learning and it utilizes a mix of labeled and unlabeled data during training. The model leverages the labeled data to learn from known patterns and utilizes the unlabeled data to generalize and infer patterns across the entire dataset. The goal of semi-supervised learning is to maximize the learning performance of the model through such newly-labeled examples while minimizing the work required of human annotators 52.

For this project, we adopted a Semi-supervised Learning approach using a pretrained neural network called xlm-roberta-base 53. This network is a multilingual version of RoBERTa pretrained on a large corpus in a self-supervised fashion. This means it was pretrained on the raw texts only, with no humans labelling them. However, semi-supervised learning requires labeled data. Therefore, it was necessary to train the network in a process called fine-tuning. The manually classified posts were normalized by removing special characters such as users and links. The data was then randomly split into two subsets using stratification for each category: 80% of the posts were allocated for training, while the remaining 20% were used for testing. This separation was performed using a seed to ensure replicability. The training subset was used for fine-tuning the network, and the testing subset was used to validate its performance on our dataset. The weighted F1-score was computed in all the categories to check the performance of the models. We executed the random split and the training three times with different seeds to ensure the good performance of the models in different partitions. In all the three iterations the model achieves similar results. In none of the categories the F1-score drops below 75%, so the precision of the models is sufficiently high. Besides, this fine-tuning methodology has shown promising results in previous studies 54. Finally, we used the fine-tunned network model (trained to apply our classification) to categorize the posts that had not been classified by hand.

### Statistical analysis

The study involved analyzing the frequency distribution of posts across various categories based on post characteristics. Percentage of posts or the median of likes and retweets in each category are reported. To compare the proportions of posts between categories, Pearson's chi-square test was utilized, yielding a p-value indicating statistical significance.

The statistical analyses were performed using the software packages STATA v16 (StataCorp).

### Ethical considerations

This project received approval from the ethics committee of Hospital Príncipe de Asturias (OE 14_2020). The ethical principles in research outlined in the Declaration of Helsinki (7th revision, 2013) were followed. This study did not directly involve human subjects or include any interventions, but solely utilized publicly available posts. Nevertheless, we have taken care not to disclose any usernames or quote posts that could reveal them, to protect their privacy.

## Results

### The most prevalent themes are the perceived low efficacy and the side effects reported by patients

Out of the total collected posts, 51,167 were deemed classifiable. Among these, 78% referred to anticonvulsants, and 24% to analgesic antidepressants. There were 1,300 posts in total containing references to both types of medications. Posts about anticonvulsants generated four times more "highly liked" posts and eight times more "highly retweeted" posts compared to analgesic antidepressants. In terms of user type, patients clearly stood out, generating two-thirds of the total posts. Furthermore, posts posted by healthcare professionals, institutions and family/friends received the most likes and retweets. (Table 1).

Regarding the content type, medical content was four times more frequent than non-medical content. 70% of the medical content posts referred to the low efficacy of the medications, while only 7% mentioned good efficacy. On the other hand, nearly 50% of the posts made mentions to the presence of side effects. (Table 1).

As for non-medical content, posts related to management predominated. Specifically, 25% of non-medical content posts mentioned difficulties in dispensing the medication at the pharmacy. Additionally, almost 15% of the posts expressed complaints about the high cost of the medication. Around 25% of the non-medical content posts requested some form of assistance. Finally, 10% of the non-medical content posts trivialized the use of the medication.

The medical posts that received the most likes/retweets were those referring to low efficacy and those mentioning the presence of side effects. In contrast, in the non-medical content posts, those discussing pharmaceutical dispensation and seeking help received the most likes/retweets (Table 1).

### Healthcare professionals are the ones who post the most medical content, while institutions post the least

All users post about medical content, but healthcare professionals, in particular, address medical topics in nearly 90% of their posts. However, healthcare institutions only post about medical topics in two thirds of their posts. (Table 2). Questions were more frequent in posts with non-medical content, and the users who asked the most questions were family members or friends. The percentage of medical posts that considered the medication to be ineffective or minimally effective was 75% for analgesic antidepressants and 69% for anticonvulsants. In other words, users' perception of efficacy was very similar in both pharmacological groups.

Similarly, the percentage of posts mentioning side effects was quite similar in both groups. Anticonvulsants had a slightly higher percentage of posts referring to problems with pharmacy dispensation. We also found a slight increase in the percentage of posts about legal issues related to anticonvulsants. Anticonvulsants had a slightly higher percentage of posts describing recreational use. Anticonvulsants were clearly the subject of a higher percentage of posts mentioning high cost. As for solidarity, antidepressants received a higher percentage of posts (Table 3).

## Discussion

Our results show that anticonvulsants have been the subject of many more posts than analgesic antidepressants, and they have generated more social media engagement. In terms of content, medical content prevailed, with a reference to the low efficacy of the medication in up to 70% of the occasions and mentioning the presence of side effects in 50% of the posts. This proportion was very similar in both pharmacological groups.

Interestingly, patients emerged as the primary contributors to the highest volume of postings, whereas the contributions from family/friends, healthcare professionals and institutional accounts elicited the greatest level of interest. This reflects that it is the consumer themselves who turn to social media to obtain information, understanding, and support from others. In several previous studies that have examined the type of user engaging in health-related discussions, it has also been found that the most common type of user is patients 43,45. Likewise, it was also noteworthy for us that despite healthcare professionals are the second group of users identified, their presence in this social media is quite limited in comparison to the highly represented group of patients. Previous studies analysing social media posts related to health topics, the proportion of involved healthcare professionals has been higher than what we found in the present study 54,55.

Pain is a challenging syndrome or symptom to treat. In fact, it is very common to combine medications to achieve adequate pain control. However, in many cases, even with combination therapy, adequate pain control is not achieved 1. In our study, we found that the most frequently repeated topic in the posts is the low efficacy of these medications. On the contrary, other studies that have also analyzed social media posts about different types of treatments reported a higher perceived efficacy by users, even in posts about diseases such as obesity or ADHD (Attention- Deficit/Hyperactivity Disorder) that are difficult to treat and also require multidisciplinary approaches and combination of treatments 42,55. On the other hand, despite being medications better tolerated than opioids, adjuvants also have side effects 56, which is reflected in the content of the posts. Nearly 37.9% of the posts mentioned some side effect, which is a higher percentage than reported in other studies analyzing posts about medical treatments 43,45.

Another prevalent topic in the posts is related to dispensing issues at pharmacies. This is concerning because previous studies have reported that this type of medication is frequently included in counterfeit prescriptions 30,57-59. Furthermore, 13% of the posts identified reservations from doctors about prescribing these medications. This finding is consistent with previous research, as an increasing number of doctors are opposed to their prescription due to agencies warning about the risks associated with their abuse 27,60-62. In fact, since 2015, a dozen countries worldwide have regulated the prescription and dispensing procedures for pregabalin, and several countries have extended these restrictions to gabapentin^63^-^65^.

2.4% of the posts promoted the medication through social media. Until now, promotion of non-opioid medications through social media had not been reported, which may reflect people's need to alleviate their pain. A potential explanation for this finding is that the posts we analyzed were published throughout 2019 and 2020, partially coinciding with the COVID-19 pandemic. Access to doctors was severely restricted in many regions of the world due to the pandemic. However, the presence of such posts may also be reflecting the existence of irregular online sales, as previously described. It is concerning that despite the efforts made by multiple institutions to regulate access to analgesics, their use without medical prescription continues to increase. Indeed, in a recent survey conducted in the United States, 17% of gabapentin users admitted to obtaining the medication without a medical prescription 56.

On the other hand, 14% of the posts referred to the high cost of the medication. This perception has also been described for other treatments for chronic diseases, such as diabetes. This fact has implications for clinical practice as it leads patients to lean towards cheaper treatments, sometimes at the expense of a diminished efficacy 66,67. Finally, it is worth noting that we have found minimal stigmatizing content, and we have not found references to suicide or any indication that these medications are currently being used for overdoses. This finding is particularly positive, yet it is crucial to monitor online discussions about drugs used as pain adjuvants, as recent studies indicate an increasing trend in the misuse of gabapentin and pregabalin 25,68-70. In France, there was a notable rise in reports of gabapentinoid abuse, increasing from 24 cases between 2010 and 2017 to 71 cases in 2018, and further escalating to a total of 117 cases in 2019 30. Additionally, the count of gabapentinoid users within the substance-using population increased by a factor of 2.6 between 2018 and 2019. Similarly, in the United Kingdom, using data from the National Programme on Substance Abuse Deaths (NPSAD), it was observed that the number of deaths related to the use of gabapentinoids increased from 8.9% in 2014 to 32.3% in 2020 71. In these cases, gabapentin and pregabalin were obtained illicitly in 38.0% of cases. Similar findings have been reported in other European countries 72,73 but also in Australia, as well as Arab and African countries 63,74,75.

### Limitations

This study has several limitations. First, it is noteworthy that Twitter users typically skew towards younger demographics, thereby potentially limiting the generalizability of these findings to the broader populace, especially to older cohorts. Indeed, individuals aged 50 to 64 accounted for a mere 21%, while those over 65 represented only 10% of the entire Twitter user base 76. Second, although our search tool has access to 100% of posts, we may have excluded posts referring to these drugs but using different keywords. Third, while we analyzed the number of retweets and likes generated by each post as an indicator of user interest in a given topic, these engagement metrics can be influenced by other factors and may not accurately reflect user interest. Fourth, we did not geolocate the origin of the posts, thus, even though the posts were written in English, we are uncertain whether they originated from countries such as the UK, the USA, or Australia, for example. Another limitation of this study is the method used to split the dataset into training and test sets. An additional experiment with a split that ensures data leakage is not occurring should be attempted to generalize the results. Finally, content analysis involves a degree of subjectivity, which we have attempted to minimize through prior training and our previous experience in this type of analysis.

## Conclusions

This mixed-method analysis of Twitter posts offers a unique and insightful approach to capturing public opinions regarding tricyclic antidepressants, pregabalin, and gabapentin. Unlike traditional survey methods, this approach mitigates social desirability biases by observing spontaneous discussions on a platform unaffected by clinical supervision and closed-ended questions. We found that the most active user type is patients, suggesting that patients frequently turn to social media to express themselves, seek information, find support, and address their medical queries. In fact, the most frequently discussed topics were efficacy, considered low in most posts, and side effects. The prevalence of medically oriented posts underscores the contemporary practice of discussing medical topics on social media, which should encourage institutions and healthcare professionals to engage actively in disseminating accurate medical information through these channels.

## Figures and Tables

**Table 1:** Number of posts generated by type of drug, type of user or type of content and number of posts that have generated at least 10 retweets or 10 likes. Percentages add up to more than 100% because there were 1,300 posts containing references to both types of medications. Relative % refers to the number of posts with likes and retweets in relation to their specific number of posts.

	Total posts			Posts with > 10 likes		Posts with > 10 retweets		
	n	%	n		Total % (Relative %)		n	Total % (Relative %)
COANALGESIC								
Total Coanalgesic	51,167	100.0%	2,108		4.10%	983		1.90%
Tricyclic Antidepressants	12,512	24.5%	423		0.83% (3.38%)	112		0.20% (0.89%)
Gabapentinoids	39,955	78.1%	1,751		3.42% (4.38%)	892		1.70% (2.23%)
User								

Patient	34,418	67.3%	1,088		2.1% (3.16%)	364		0.7% (1.06%)
Family / friend	5,104	10.0%	196		0.3% (3.84%)	161		0.3% (3.1%)
Healthcare professional	6,830	13.3%	528		1.0% (7.73%)	191		0.4% (2.8%)
Institution	4,815	9.4%	296		0.5% (6,15%)	267		0.5% (5.54%)
Content								
Total Medical content	40,767	79.6%	1,637		3.2% (4.02%)	405		0.7% (1%)
Efficacy	31,914	62.37%	1,269		2.48% (3.98%)	295		72.8% (0.92%)
- None or little efficacy	28,863	56.41%	1,165		2.28% (4%)	277		68.4% (0.96%)
- Good efficacy	3,051	5.96%	104		0.2% (3.4%)	18		4.4% (0.59%)
Side effects	19,398	37.91%	893		1.75% (4.6%)	285		70.4% (1.47%)
Total No medical content	10,400	20.3%	471		0.9% (4.52%)	578		1.1% (5.56%)
Management issues	6,439	12.58%	284		0.56% (4.41%)	147		0.28% (2.28%)
- Pharmacological dispensing	2,679	5.24%	93		0.18% (3.47%)	58		0.11% (2.16%)
- Medical prescription	1,368	2.67%	69		0.13% (5%)	36		0.07% (2.63%)
- Commercial	1,244	2.43%	55		0.11% (4.42%)	23		0.05% (1.85%)
- Legal issues	1,148	2.24%	67		0.13% (5.84%)	30		0.05% (2.61%)
Economic aspects	1,477	2.89%	57		0.11% (3.86%)	31		0.05% (2.1%)
Refers to solidarity	2,542	4.97%	101		0.2% (3.97%)	442		0.86% (17.4%)
Trivialization	1,091	2.13%	64		0.13% (5.87%)	14		0.03% (1.29%)
- Humour/Joke	389	0.76%	23		0.04% (5.91%)	5		<0.01% (1.29%)
- Recreational use	578	1.13%	36		0.07% (6.22%)	8		<0.01% (1.4%)
- Song/Poetry/Book	124	0.24%	5		<0.01% (4%)	1		<0.01% (0.8%)
Query								
Refer to a Query	4,200	8.2%	131		0.2% (3.11%)	52		0.1% (1.24%)
								

**Table 2:** Number of posts according to publication content and user type. The P value was <0.001 for all comparisons.

	Patient		Family/friend		Healthcare professional		Institution	
	N posts	%	N posts	%	N posts	%	N posts	%
Medical content	28,084	81.6%	3,743	73.3%	5,877	86.0%	3,063	63.6%
Non-medical content	6,334	18.4%	1,361	26.7%	953	14.0%	1,752	36.4%
**TOTAL**	34,418	100.0%	5,104	100.0%	6,830	100.0%	4,815	100.0%
**P value <0.001**								

**Table 3:** Distribution of drug categories according to medical and non-medical content posts. The P value was <0.001 for all comparisons.

CATEGORIES	Tricyclic Antidepressants		Gabapentinoids		P-value
	N posts	%	N posts	%	
	Medical content				
Total Medical Content	11,504		30,471		
None or little efficiency	8,637	75.1%	21,084	69.2%	P<0.001
Good efficacy	750	6.5%	2,448	8.0%	
Side effects	5,106	44.4%	14,768	48.5%	P<0.001
	No Medical content				
Total No Medical Content	1,008		9,484		
Total Management	472	46.8%	6,037	63.7%	P<0.001
* -Pharmacological dispensing*	155	32.8%	2,533	42.0%	
* -Medical prescription*	109	23.1%	1,280	21.2%	
* -Commercial*	131	27.8%	1,127	18.7%	
* -Legal issues*	77	16.3%	1,097	18.2%	
Refers to a high cost	63	6.3%	1,425	15.0%	P<0.001
Refers to solidarity	287	28.5%	2,258	23.8%	P<0.001
Total Trivialization	172	17.1%	924	9.7%	P<0.001
* -Humor/Joke*	56	32.6%	336	36.4%	
* -Recreational use*	76	44.2%	504	54.5%	
* -Song/Poetry/Book*	40	23.3%	84	9.1%	
